# The effectiveness of primary health care reforms in Greece towards achieving universal health coverage: a scoping review

**DOI:** 10.1186/s12913-021-06678-9

**Published:** 2021-07-01

**Authors:** Thanos Myloneros, Dikaios Sakellariou

**Affiliations:** 1 Formerly London School of Hygiene and Tropical Medicine, Keppel Street, London, WC1 7HT UK; 2 Cardiff University, School of Healthcare Sciences, Eastgate House, Newport Road 35-43, Cardiff, CF24 0AB UK

**Keywords:** Greece, Primary health care, Universal health coverage, Healthcare access, Health reforms

## Abstract

**Introduction:**

Between 2010 and 2018, Greece implemented an Economic Adjustment Programme and underwent a series of extensive reforms, including in the health sector. We conducted a scoping review to examine whether the Primary Health Care reforms during that period assisted the country in moving towards Universal Health Coverage.

**Methods:**

We performed a review of the literature on the following databases: Scopus, PubMed, Epistemonikos, Web of Science, and Google Scholar, including published research articles and grey literature. Findings were synthesised thematically, using the World Health Organization Universal Health Coverage dimensions: population coverage, service coverage, and financial protection.

**Results:**

Forty-four documents were included in this review. Out of these, thirty-eight were research-based (thirty-three qualitative, two quantitative, and three mixed design studies), two grey literature, and four legislative bills. The evidence suggests that despite the systemic interventions addressing longstanding distortions, population coverage, service coverage and financial protection have not significantly improved.

**Conclusions:**

This review suggests that Primary Health Care reforms in Greece have not managed to substantially improve Universal Health Coverage, although some positive steps towards that direction have taken place with the establishment of community-based multidisciplinary health teams. Before further interventions are implemented, an evidence-based monitoring and evaluation mechanism is necessary in order to clearly evaluate their effectiveness and progress.

**Supplementary Information:**

The online version contains supplementary material available at 10.1186/s12913-021-06678-9.

## Introduction

### Background

Ever since the Declaration of Alma-Ata in 1978 [1], Primary Health Care (PHC) has become the cornerstone of any sustainable health system [2]. In the following decade and as a response to the emphasis on PHC, Greece created rural health centres as part of the National Health System (known as ESY) but failed to develop strategies for comprehensive country-wide PHC services [3]. This allowed multiple distortions to thrive and accumulate, such as fragmentation of services and funding, excessive reliance on specialist care, lack of care pathways, and supply-induced demand with consequent high out-of-pocket payments (OOP) as percentage of total health spending, compared to the rest of the EU-27 countries [4]. These distortions made the health system susceptible to socioeconomic fluctuations [5].

The 2008 global financial crisis affected Greece in multiple ways and had a big impact on the health sector [6–9]. In 2010, the country entered a bailout programme known as Economic Adjustment Programme [10], which lasted until 2018 and was implemented through three consecutive Memoranda of Understandings (MoU), signed between the Greek State and the International Monetary Fund, the European Commission, the European Central Bank and,at a later stage, the European Stability Mechanism. These MoUs came with a prerequisite but negotiable package of reforms, comprised mainly by cost-containment policies. One of the major reforms was related to the health sector, but the horizontal fiscal consolidation approach applied across the other sectors, complicated the inherent weaknesses of the health system [5, 11]. Guidance received by the World Health Organizatiom (WHO) made it clear to the Greek government that in order to improve Universal Health Coverage (UHC) [12] the establishment of a comprehensive PHC network was necessary [13].

PHC is critical for improving all dimensions of UHC, which include population coverage, service coverage, and financial protection, through reaching all people, including marginalized and disadvantaged populations, increasing access to quality services, medicines and vaccines, and reducing household expenditure on health [14, 15]. Particularly, PHC focuses on tackling health and socioeconomic determinants through community-based services, which in most cases is the only way to reach marginalized populations and identify vulnerable groups [16]. There is considerable evidence that health systems with strong people-centred, continuous, comprehensive, and coordinated PHC are more sustainable and have better health outcomes [17]. Furthermore, low household expenditure on health is strongly associated with the use of preventive approaches and health promotion, in contrast to the more expensive retroactive disease-management approach [18]. Avoiding escalation of health issues can be achieved through the provision of community-based care, including preventive services that only primary care can achieve [19].

### The reforms

Prior to the financial crisis, PHC in Greece was provided through the ESY, Social Health Insurance (SHI) polyclinics (mainly staffed with specialists), contracted physicians and private practices. During the first phase of reforms, the SHI schemes were merged to form the National Organisation for the Provision of Health Services (EOPYY) [20], covering the majority of the population. For the transition phase, EOPYY continued to be a provider, but in the second phase of reforms the SHI PHC services were integrated with ESY, to form the national PHC network (PEDY) [21]. In the third phase of reforms, geographical areas of responsibility were introduced called “PHC sectors” [22], within which both public and private providers could form local networks and provide community-based care in two levels; The first level includes the existing rural solo practices, the contracted family doctors and the new Local Health Units (TOMYs). These units are staffed with multidisciplinary health teams consisting of family doctors, nurses, health visitors, social workers, and administrative staff and their aim is to address major health-related issues at the community level, reduce avoidable hospitalizations, provide patients with care as close to their homes as possible, and address public health issues at their roots by targeting behaviour and risk factors. The second level of care includes the referral Health Centres providing primary, ambulatory, diagnostic, acute and emergency out-of-hour care [5], and the contracted specialist and diagnostic private services. The new model was designed with the ambition to evolve into an integrated health care model [23].

While conceptually these reforms embody the fundamental principles of PHC, their effectiveness has not yet been evaluated in a comprehensive way. The main aim of this review is not to assess the PHC services in Greece overall, but to identify through the available literature whether the PHC reforms in Greece during the period of the Economic Adjustment Programme, had any impact on the UHC dimensions of population coverage, service coverage and financial protection.

## Methods

We conducted a scoping review of the literature in June–July 2020 on the following databases: Scopus, PubMed, Epistemonikos, and Web of Science for the main search in English terms and Google Scholar for the supplementary search using both English and Greek terms. We used the PRISMA-ScR (Preferred Reporting Items for Systematic reviews and Meta-Analyses extension for Scoping Reviews) guidelines [24] to report the review process.

### Research question

The scoping review sought to examine whether the PHC reforms assisted Greece in moving towards UHC by improving its dimensions. The review question was structured based on the PICO Framework [25]:

- Population: Residents and citizens of Greece.
- Intervention: Access to Primary Health Care services.
- Comparison: Before and after the reforms.
- Outcome: Expansion of the three dimensions of UHC.

### Search concepts and terms

The search was focused around the three main concepts of the question: *Primary Health Care*, *Universal Health Coverage* and *Reforms*. The search included terms both in the English and in the Greek language. For brevity, we only include the English language terms in Table 1, but the full list is available upon request.

**Table 1:** Search concepts and terms

Key concept	Search terms
Primary Health Care	Primary Health Care
	PHC
	Primary Care
	PC
	Family medicine
	TOMY
	PEDY
	EOPYY
	Integrated care
	Integrated / Integration
Universal Health Coverage	UHC
	Access
	Coverage
	Financial protection
Reforms	Economic Adjustment Programme
	MoU
	EC
	Reform / Reforms

### Search strategy

The search was divided into two parts, one with the English and one with the Greek terms. For the first part, the term “Primary Health Care” and its alternative “Primary Care” were used interchangeably in all areas. As for Universal Health Coverage, the term was broken up to its components, adding also the terms “integrat*” to include indicators of integration. Table 2 shows the general search string. For an example of the exact search string for each database see supplementary material A.

**Table 2:** General search string

[**TITLE-ABSTRACT-KEY WORDS** (Primary Health Care OR Primary Care OR PHC) ***AND*** (Greece OR Greek)] ***AND / OR*** [**ABSTRACT–KEY WORDS** (Service*) OR (Coverage) OR (Access) OR (Reform*) OR (PEDY) OR (TOMY)] ***AND / OR*** [**KEY-WORDS** (Health Centre*) OR (PEDY) OR (TOMY)]	

### Eligibility criteria

The search results were filtered through the following inclusion criteria prior to reviewing. Only papers in English or Greek were included, published from 2010 until June 2020. The period was chosen because the Economic Adjustment Programme started in 2010 and PHC reforms were initiated in 2011. Type of documents included journal articles, research documents (all designs), reviews, and health policy reports. To be eligible, articles had to include information related to aspects of PHC in Greece through a variety of dimensions with focus on access, coverage, quality, spectrum of services, inclusion, user satisfaction, governance, human resources and integration, that could be linked with policy making within the given timeframe. Clinical trials or disease-oriented research papers in PHC environment were excluded.

### Data extraction and synthesis

We extracted relevant data from the findings and discussion sections (and other sections, especially for non-research sources) of the included articles. We performed a narrative synthesis guided by the three dimensions of UHC: population coverage, service coverage, and financial protection [12].

## Results

### Characteristics of included articles

**Fig. 1:**
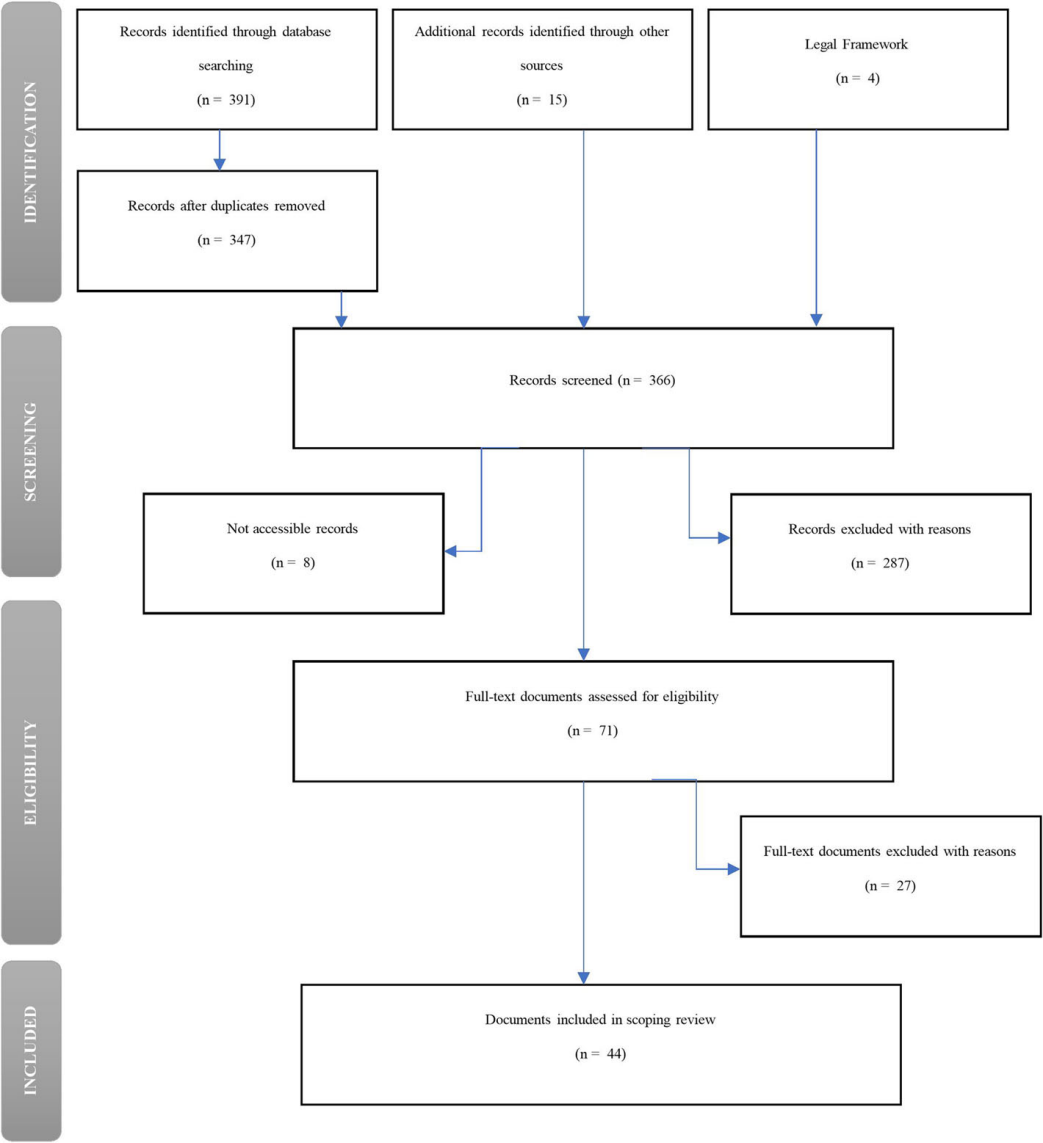
PRISMA diagram

The database search returned 391 documents. After duplicates were removed, the titles of 347 documents, and where necessary their abstracts, were screened for inclusion along with 19 documents that were identified through other sources, including 4 legislative documents. Out of the 366 records screened, 8 were not accessible in full text and 287 were excluded with reasons. The remaining 71 full-text documents were assessed for eligibility and 27 were excluded. In total, 44 documents were included in this scoping review (see Fig.1 for the detailed selection process).

Out of the final 44 documents, 38 were research-based: 33 were based on qualitative design, 2 on quantitative, and 3 on a mixed design. We also included 2 documents classified as grey literature and 4 parliamentary laws. Laws aside, the majority of the documents included (27 out of 39) were published in the second half of the decade and 10 in 2019. For the full list of reviewed documents please see supplementary material B. All included documents (apart from the laws) were critically appraised. No articles were excluded upon screening process. The Mixed Methods Appraisal Tool (MMAT) [26] was used for published literature and the AACODS [27] checklist for grey literature (see supplementary materials C, D, E, and F).

### Narrative synthesis

#### Population coverage

While prior to the reforms a part of the rural population could access health centers and practices, in urban areas PHC was delivered mainly by ambulatory clinics and private specialists rather than GPs, of which a small percentage contracted with the SHI schemes [28]. Even though overall satisfaction for PHC services was relatively high and users understood the important role of a primary care physician, doctors, at least in rural areas, identified significant gaps in the provision of PHC, among which notably the lack of community nurses [29–31]. However, the evidence suggests there is a conflicting perception of what PHC entails, with further divergence regarding its effectiveness, especially between users and providers [31].

Until the completion of the SHI merge under EOPYY in 2014, population coverage and access to care were reduced significantly, especially in urban areas, for three main reasons: loss of insurance entitlements due to increased unemployment (up to 27.4%) [32], excessive reduction of human resources due to doctors moving to private sector or abroad and reduction of services [33]. Shrinking public health expenditure caused a reduction of human resources, supplies, and services [34] further burdening the already limited resources [29], thus reducing population coverage and leading service users towards the private sector. One consequence and strong indication of the ensuing gaps in service provision, especially in urban areas, was the establishment of solidarity PHC clinics by citizens as a response to the health system inefficiency. These clinics provided basic primary and emergency care, mostly to people with no health insurance, including refuges and migrants. A survey among 92 solidarity clinics, reported a 10% or more increase in the number of patients attending from 2013 to 2014 [35].

At the peak of unemployment followed by a steady increase of unmet healthcare needs [36], law 4238/2014 attempted to reorganize PHC. Although it attempted to resolve structural distortions, significant difficulties were met during implementation [37] mainly because it was rather fiscal-driven within the overall mindset of economic reforms. While the integration of the former SHI polyclinics into the newly-established PEDY network under the ESY aimed at defragmenting services, instead it led to an association of PHC with ambulatory specialist care. Furthermore, it was met with resistance from the contracted doctors who were working in the SHI polyclinics while having their private practices at the same time. These doctors were called to decide whether to work for the public or for the private sector, leading many to opt-out from their public-sector contracts, thus affecting population coverage [33]. Moreover, the integration of services did not address the issue of the unequal distribution of health professionals between urban and rural areas and failed to introduce the family physician as a mandatory first point of contact [3, 33]. Later studies revealed that the number of specialty doctors and their relative distribution among specialties remained almost unchanged between 2010 and 2015 [34, 38].

In 2015, 1 year after the second reform, overall satisfaction in PHC was relatively low (48.6%) and most participants reiterated the need for a new approach, whereas the majority of the participants (81.8%) reported a preference for a family physician that would guide them to specialist services [39]. The latter is supported by other studies too, which report that service users, and especially those with low health literacy, expect to be guided by doctors [40].

Furthermore, the increasing arrival of refugees and migrants from that year onward, caused additional pressure to the health system and increased the burden of disease among marginalized groups. Stigmatization, exclusion, and powerlessness have been identified as the main barriers for poor access to healthcare for this population [41]. Administrative and structural barriers especially with the asylum process caused further exclusion [37]. Recent evidence indicates that migrants face significantly more barriers in accessing health services than non-migrants [42], especially those with chronic diseases and in need for medicines [43].

In 2016, law 4368/2016 [44] granted access to public healthcare services to the uninsured population,, and the following year, law 4486/2017 [22] introduced a new PHC model [45]. This aimed to steer care back to the community, with a focus on prevention, health promotion, and integrated care [46], also providing care for free to those with no insurance. The initial phase of the new reform included the planned roll-out of 239 multidisciplinary units, the TOMYs, within a timeframe of 2 years, but as of June 2019 the roll-out had paused at approximately half of the original target, with 127 TOMYs [47–55]. In addition to that, the potential registration capacity of the TOMYs based on their staffing at the end of 2019 was 650,250 citizens; however, at that time there were just about over 400,000 registered citizens [55]. On the upside, there existed an increasing trend to register with family doctors and health care teams, in contrast with the previous state of no registrations at all, which is a slight improvement in population coverage in terms of actual PHC services [55]. Unavailability of GPs and inadequate premises are largely considered to be the main reasons that not all planned TOMYs started to operate [5, 55]. The evidence reveal that there was only a slight increase in GPs amidst the reform, from 3.4 per 10,000 inhabitants in 2017 to 3.6 in 2018, which is still the lowest in Europe [56]. This might be partly attributed to dissatisfaction with their level of income [57], especially in rural and remote areas.

#### Service coverage

Ambulatory and PHC services in Greece are accessible for a wide range of preventive procedures including blood tests, early diagnosis of chronic conditions and immunization under a national immunization programme, but administration of booster doses is often delayed [3]. This might indicate discontinuation in services and difficulty to follow up, for a variety of reasons, like the lack of medical records. Despite the ability of PHC in performing preventive and public health interventions, Greece reports one the highest rates of chronic diseases among the EU-27, with cardiovascular diseases and lung cancer being the leading causes of death, while mortality from diabetes and chronic respiratory conditions have increased over the last two decades [58–60]. Chronic conditions are not addressed adequately and integrated care is in an embryonic stage [61]. Even though approximately 25% of deaths are attributed to behavioral and lifestyle risk factors (including tobacco use and obesity), which could be addressed at the primary care level [37], there is still a long way ahead towards actual integration and chronic disease management. Despite the reforms, levels of integration are still quite low [62] especially with public health services and significant gaps in health promotion and preventive services have been identified [63, 64].

Prior to 2011, social insurance schemes covered almost the entire population, but by 2016 25% of the population lost their right to use EOPYY-financed health services [3]. When insurance funds merged under EOPYY in 2011, the package of services was rationalized and standardized. However, instead of investing on equal access to primary care services and family doctors, a big portion of the fund’s expenditure was given retroactively to hospitals, diagnostic services, and pharmaceuticals [28, 58]. A 2015 survey showed that the most common reasons for utilizing PHC were acute problems, drug prescription, and routine checkups, with only half the participants reporting adequate consultation time with the doctor [39]. The majority of the consultations were done within 10 min, which is a very short timeframe to perform preventive medicine and address behavioral risk factors. In the same study, 25% of participants reported low quality of healthcare services [39]. Later evidence revealed services being reduced to the bare minimum, with inadequate consultation time and it was highlighted that health literacy and support for self-management could not be achieved with such short consultation times [40].

In populations where there is low access to healthcare and even more so among vulnerable groups like older people with multimorbidity, self-management seems to be the most relevant modality [64] and a core element of integrated models. Chronic and palliative care are often either provided by relatives or informal carers, when at the same time health literacy which could support carers or self-management is at a significantly underdeveloped stage [40]. Consultation time for people with multimorbidity is not adequate, older people find the system too complex, and the one-point contact that could be a family doctor or other professional, is often missing [40]. People-centredness and integration are re-emerging issues brought up by users themselves much before the onset of the crisis [31]. Vulnerable and marginalized groups did not usually receive prevention and health promotion services [64]. It is noteworthy that the most frequent requests among PHC users in the recent years was the increase in the number of public health units [65]. Service users report that family medicine is necessary [31], indicating that citizens are not against a central role of PHC.

In regards to the TOMYs, the more frequently provided services include promotion of population health, planned adult and child health care, elderly health care, multi-morbidity monitoring, development of interventions and actions to promote health in the community, whereas services that need further development include public health services (including vaccination services), home-based healthcare, post-hospital care and rehabilitation [55]. A survey among TOMY users, indicated relatively high satisfaction, especially regarding the quality of medical and nursing care [66]. It is noteworthy that the quality of nursing care had high mean scores, which could indicate the gradually increasing trust of people to nurses in PHC and an expansion of their responsibilities. Although there seems to be a reorientation towards preventive services and community outreach as depicted in the 2017 law [22], it still remains to be assessed whether the actual practice corresponds to the legal framework [5, 66]. The overall framework of the job description does not automatically allow implementation, as operational and clinical guidelines have to be developed and introduced [37, 67]. Surveys among the employees suggest though that healthcare teams are happy that they can use their flexibility and autonomy to formulate community outreach actions [55].

Considerable differences, however, were found between facilities in their orientation towards acute or chronic care, with the most efficient ones focusing on prevention and chronic disease management [68]. Overall, disease management appears ineffective and there is no community outreach, whereas only few people receive screening services [5]. Even though evidence shows that GPs agree on the importance of screening in improving care, GPs older than 50 years of age, those practicing for more than 15 years, and GPs working in private sector, are less likely to comply with screening recommendations [63]. In practice the situation is more complex, as few clinical guidelines are in place in most of the PHC units and medical records are being kept internally [69]. Almost 9 out of 10 family doctors in the TOMYs report that they use a package of 13 General Medicine Guidelines/13 PHC Protocols provided by the Ministry of Health, along with an operational guideline called “Handbook for the Operation of Local Health Groups” [55]. It appears though that care for chronic patients and frail people along with promotion of self-management, need to be improved as does disease management through recorded clinical governance and data analysis [70].

Other limitations at achieving operational integration include absence of an organized referral system that could support the handling of emergencies [71], patient pathways, and an established social and community care system [37, 72, 73]. Electronic health and telemedicine have not been developed, despite the geographical dispersion of islands and remote areas and the high concentration of refugees especially on the islands of the North-Eastern Aegean, with limited provision of healthcare services [42, 43]. Consequently, the majority of the refugee population seek care at the emergency departments of hospitals and specialist care and not in PHC, whereas preventive services, with a few exceptions for certain communicable diseases and vaccinations, are not provided almost at all [43].

Apart from TOMYs, which have social workers in their teams, evidence suggest that PHC rarely supports mental health services, at least not in a coordinated manner. The number of people visiting PHC during the financial crisis of 2008–2018 due to mental health concerns increased [74], exacerbating already severe gaps in mental health service provision [75]. Communication and collaboration between primary care and mental health services is rather ineffective and professionals on both ends are rarely trained to refer people on to other services [76]. It has been suggested that the inclusion of psychiatrists in PHC might decrease the social stigma around mental health, increase population access to mental health services, and improve the detection and management through multidisciplinary involvement [77]. Overall, evidence indicates that service users are not involved in health decision making, stakeholders are not trained adequately to understand and promote integrated care, and the coordination of care is absent [67].

#### Financial protection

The case of Greece shows that linking healthcare entitlements with employment and contributions can significantly increase OOP for healthcare during an economic crisis. By 2016, approximately 25% of the population was uninsured thus not having access to healthcare services [28] and although law 4368/2016 [44] granted access to public services, the uninsured still had no access to services financed by EOPYY, including the EOPYY contracted physicians. In 2010, OOP was 28.6% of the health spending whereas in 2018 increased to 36.44% [4]. Catastrophic health spending appeared to decrease, but unmet needs increased. Evidence shows that 7.2% of households in Greece had experienced catastrophic health spending in 2010, a percentage that increased to 10.5% in 2015 and slightly dropped to 9.7% in 2016. The average OOP is much higher than expected, while catastrophic health expenditure increased particularly in the poorest quintiles of the population [78].

In 2014, EOPYY contracted doctors on a fee-for-service basis with a limit of 200 visits per month, paying the doctor 10€ per visit [33]. The limit was reached within few days and after that people had to pay using OOP [3, 5, 28]. Sometimes service users paid informally to avoid searching for a doctor who had not reached their visits and prescription limits [5]. This supply-induced demand for specialist services rather than preventive medicine, increased private expenditure and OOP, both through co-payments and informal payments. Until 2016, 44% of OOP among households who experienced catastrophic health spending were for medicines particularly among the poorest quintiles [78]. Furthermore, in regards to PHC services, outsourcing the appointment system for the contracted physicians, passed on the cost to the caller/patient, with prices ranging from ﻿€0.95 to €1.65 per minute [5].

Medicines and pharmaceuticals – with few exceptions – are not provided at PHC facilities. Patients receive the prescription and pick up the medicines at private pharmacies. An e-prescription system was initiated in 2010 and 2 years later prescription guidelines were also introduced. Most prescription medicines in Greece are dispensed with a fixed co-payment which varies between 0% for people exempted (uninsured, vulnerable etc) and 25% [5]. Service users also have to pay the difference between the price reimbursed by EOPYY and the retail price [5, 58], which might explain the reason why the share of OOP for pharmaceuticals is the largest [58], despite the significant pharmaceutical expenditure of the organization [33]. The amount people have to pay OOP therefore varies depending on the medicine purchased [3]. The negative effect of this co-payment policy is magnified when medicine prices are relatively high (e.g. due to inadequate regulation) and when doctors and pharmacists are not required or do not have incentives to prescribe and dispense cheaper alternatives. Overall, the household expenditure for pharmaceuticals and ﻿the average proportion of co-payments per month has increased from 9% in 2009 to 30% in 2016 [5, 28].

In addition to co-payments for prescribed medicines, OOP for medicines may arise through the purchase of over-the-counter medicines [3]. It is worth mentioning that the profit margin for pharmacies for over-the-counter medicines and non-reimbursed prescribed pharmaceuticals is around 35% [3]. Evidence also suggests that in order to avoid paying both for the doctor visit and the medicine, service users sometimes prefer to obtain prescription medicines over the counter, which means they have to pay the full cost [5]. This is facilitated by weak enforcement of regulation governing the dispensing of medicines [78] and by the availability of 216 out of 1582 (in 2016) over-the-counter medicines in stores other than pharmacies [3].

Disadvantaged and vulnerable groups, like migrants and unemployed people, reported financial barriers mostly for pharmaceuticals, which indicates barriers in access to PHC, exclusion from benefits, and unprescribed over-the-counter self-medication [42, 43]. In response to this, citizens organized PHC clinics in the form of solidarity outpatient clinics providing preventive, chronic and emergency healthcare to, mostly, uninsured people and migrants [35]. It is worth noting that even though disadvantaged groups like the uninsured, chronic patients, pensioners, and migrants find that healthcare is too expensive, they tend to prefer, where possible, the private sector over the free public one, with the perception of superior quality and timely access [40, 79].

## Discussion

The aim of this scoping review was to identify through available literature, in what extend the PHC reforms in Greece that were implemented during the period of the Economic Adjustment Programme, have impacted the three dimensions of UHC. A full evaluation of PHC services was beyond the scope of the review. The examination of the scarce evidence identified from the given timeframe, suggests that the PHC reforms were not significantly effective in improving UHC, they created however the framework within which the important next steps can be taken. The legal framework developed during that period and the development of a person-centred approach in PHC, show a distinctive shift towards improving UHC [21, 22].

Between 2010 and 2018 successive governments implemented several reforms, often moving to the next one without the previous having been thoroughly evaluated [5] and with an apparent reform fatigue among the implementing authorities. Even though the political risk was shifted to the supervising institutions which imposed these measures [80], horizontal measures and austerity cuts (per capita expenditure between 2008 and 2013 dropped by almost 10%) increased inequalities especially among vulnerable groups [81, 82]. The 2017 PHC reform was linked to the UHC and aimed to lead to a more streamlined health service, with higher integration of services, increased accessibility, and limited financial risks for the population [22]. This study demonstrates that while some improvements were made, many problems remain or even got worse across all dimensions of UHC, leading to compromised access to care, gaps in care provision and low quality of care, exemplified by high OOP and high risk of catastrophic payments, particularly affecting people with lower income.

Evidence shows that people met severe barriers in accessing health services during the crisis [28, 83]. The rather low number of PHC workforce and especially GPs [34, 56] along with the rural-focused distribution of Health Centres [54] undermined access. The main reform among those examined, the establishment and development of TOMYs, has some important strengths, like the person-centred approach, the introduction of multidisciplinary teams and provision of services close to the community, but has also some weaknesses, including incomplete integration and continuation, limited interconnectivity and moderate population coverage [55]. The opening of just 127 new units expanded the population coverage locally, improving access only for those registered in them. It would take approximately 850–900 such units to cover 85% of the whole population. Also, the TOMY network is geographically concentrated in the mainland of Greece while the islands - with the exception of Crete - are poorly covered [47–53, 55]. Coverage of the entire population would demand a significant increase of GPs [34] and until then it seems that a large part of the urban population will continue to substitute primary care with outpatient care in hospitals or specialist care in the private sector [79]. Furthermore, Greece has one the lowest number of nurses among the EU27 countries, especially in PHC, with 3.4 nurses per 1000 inhabitants [84], which implies that their role is underestimated and the state has failed to deploy a variety of health professionals to provide PHC services other than doctors.

Interlinked with the dimensions of population and service coverage, are the principles of equity of access, quality of services, and overall accessibility [85, 86]. The evidence suggests that even in cases that services are available and the population is covered, access is not always equitably distributed. People with chronic or mental health issues, people with disabilities, refugees and migrants experience compromised access to healthcare [37, 40, 43, 87]. Attending to these groups could significantly improve priorities and quality for all service users [69]. Migrants and refugees are excluded mostly due to financial and cultural barriers [88], although they do have a legal right to access in public services if they have an insurance number or their asylum paper number [42, 43]. Mental health patients and people that acquire care at home or constant care are usually excluded from primary care services and when they do have access it is rarely for preventive services [74, 77].

Similarly, oncology patients, for example, do not receive services at home or at the community level, but only in hospitals. While home care visits to people with chronic illnesses were performed in remote areas by healthcare staff, in cases where community health centers were close to a hospital, they tended to refer to the hospital [89], which implies that distribution of human resources is unequal and affects population coverage [62].

The establishment of EOPYY [20] and the supplementary integration of SHI providers into ESY [21] addressed to some degree the fragmentation of services, without though achieving continuity and comprehensiveness [3, 5, 37]. Fragmentation has been reduced, but the governance mechanisms remain complex [46, 72] with an ageing health workforce resilient to change [5, 34]. Severe gaps exist in prevention and health promotion [64] as part of the vicious cycle of over-specialization and hospital-centred services. Part of these shortcomings is the significantly low level of health literacy and people empowerment, mostly due to short consultation time and lack of workforce training [40]. The lack of a referral system and care pathways stands in the way of coordination across levels [5, 37, 62] and undermines the role of PHC in people-centred healthcare. Operationally, TOMYs have shown signs of how integrated care could be introduced [46, 66], but concrete steps remain to be taken [37].

For example, screening for the early detection of certain chronic illnesses, such as cardiovascular disease, cancer, mental disorders, and dementia, is not included in a coordinated manner and with specific guidelines [73]. Another example is that while the ratio of smokers is high, there are no smoking cessation services in the community, with available services provided through local initiatives, relying on people seeking out services [72]. Some targeted prevention services do exist, for instance related to women over 50 years old (mammography) and adults over 65 (influenza vaccination) [64].

Finally, there is no evidence linking OOP directly to PHC but rather to the whole spectrum of services [4, 58, 78]. However, we can extrapolate that the reforms improved financial protection marginally and only for those registered to a family doctor or PHC unit, as the extended opening hours of TOMYs, the proximity to citizens’ residence and the ease of arranging appointments improved accessibility to free services [55]. Even though the 2016 law [44] granted access to public health services for the uninsured, including undocumented migrants, private spending for health did not decrease [4, 58]. This happened mainly because the reforms focused on the provision of hospital-based services and did not address the root causes and inequalities in accessing healthcare [5, 78] and uninsured people have access only to public services, whereas they have to pay for contracted services.

### Strengths and limitations

The main strength of the review is that the information gathered did not present conflicting findings; on the contrary, the emerging themes across the reviewed documents revealed common patterns of systemic issues, enhancing the reliability of the findings. However, this might also be a reflection of the rather narrow PHC spectrum under examination from the available literature, mainly focusing on the provision of services. To address this, we attempted to extrapolate information from a diverse set of literature, in both English and Greek, and link the core dimensions of UHC to their results. Finally, we would like to underline the possibility of bias in the analysis of the findings due to one author’s involvement in the design, planning, and implementation of the TOMYs reform. On the other hand, that is also a strength, as the actual experience of participating in the reform provides insights and the tools to identify the key issues and challenges.

## Recommendations

Based on the findings and guided by the WHO-European Framework for Action on Integrated Health Services Delivery, which “*anchors actions in the same principles of a primary health care approach for people-centred health systems*” [90, 91], we recommend the following PHC-centred actions towards achieving UHC, prioritized by importance and grouped in the four domains of the framework.

### System enablers

1. **Incentivize and increase the PHC workforce**. Strategic choices based on population needs should lead the health professional inflows, with focus on increasing the numbers of GPs and nurses.
2. **Ensure enough time for consultation**. Quality PHC revolves around building trust between the health professionals and the patient, in order to change behavioral risks, reduce unnecessary treatment and improve health literacy. That cannot be achieved within short consultation timeframes.
3. **Establish an across-all-levels electronic health record**. Referral mechanisms, patient pathways, chronic care models and public-health-oriented design of care cannot be accomplished without a comprehensive health record in place to link services vertically and horizontally.
4. **Reduce or eliminate co-payments related to PHC**. This can be achieved through horizontal interventions, like increase of public health expenditure and earmarked budgets for PHC and targeted actions like minimizing or eliminating co-payments for medicines prescribed by family doctors or PHC professionals.

### Service delivery processes

5. **Update primary care curricula and introduce continuous professional development**. Properly trained health professionals can plan actions to tackle health determinants based on evidence, adjusted for the local context, living conditions, and vulnerability among other factors. The ever-changing evidence on improving quality of care requires the continuous professional development of the workforce.
6. **Expand the skill-mix of primary health care services** to strengthen the multidisciplinary approach, with a broader spectrum of responsibilities for nurses, midwives, and public health professionals. In this way flexible peer networks can be developed within the communities organizing care around the person and exchanging experience.
7. **Integrate public health and mental health services**. People’s health needs are not separated, with environmental, population and mental health problems often co-existing. Integrated people-centred care has to be designed and tailored around those needs in a dynamic way, flexible enough to change according to these needs.

### Change management

8. **Develop evidence-based planning, implementation, monitoring, and evaluation mechanisms**. Monitoring mechanisms based on updated evidence are necessary for the implementation of all actions and interventions.
9. **Adopt a whole-of-society approach**. Reforms can be sustainable only through inclusion, consensus and shared decisions. Continuation is vital for health policy when results are long-term and this can be achieved through society ownership and coalitions across all levels.

### Populations and individuals

10. **Strengthen health literacy and social inclusion processes**. Enhancing health literacy to support service uses, families, and carers including them in the decision-making process can contribute to the sustainability of the reforms and promote accountability.

## Conclusions

The impact of the financial crisis of 2008–2018 to the health of the Greek population and to the healthcare system have been well-documented. This review aimed to explore the extent to which the instigated PHC reforms met their aim to improve UHC, based on the available literature. Overall, the PHC reforms did not have the same impact across the population. Population coverage increased only locally due to unequal distribution of services both geographically and socially. The services dimension slightly improved for service users with access to the new PHC units and family doctors, but community outreach, public and mental health services, home care, integration, continuity and comprehensiveness need strengthening. Issues remain regarding financial protection, especially among vulnerable and disadvantaged population groups, like people with chronic illnesses, disabilities, migrants and refugees and uninsured. In order to achieve UHC, more concerted efforts are required to enable access to high quality, affordable, and appropriate healthcare. Concluding, the evidence suggests that the reforms were not significantly effective in improving UHC, however they introduced concepts that created the necessary framework for the next steps. It remains imperative that these next steps towards developing stronger PHC must be evidence-based. The weakness of a yet underdeveloped PHC can be turned into an opportunity by using the experience of other countries and studying the lessons learned.

## Supplementary Information

**Additional file 1.**

## Data Availability

The datasets generated and/or analysed during the current study are publicly available.

## Abbreviations

EOPYY: *Greek abbreviation for* National Organization for the Provision of Health Services
ESY: *Greek abbreviation for* National Health System
EU: European Union
GP: General Practitioner
MoH: Ministry of Health
MoU: Memorandum of Understanding
OECD: Organisation for Economic Co-operation and Development
OOP: Out-of-pocket payments
PEDY: *Greek abbreviation for* National Primary Care Network
PHC: Primary Health Care
SHI: Social Health Insurance
TOMY: *Greek abbreviation for* Local Health Unit
UHC: Universal Health Coverage
WHO: World Health Organization
