# Supplementary material for: The effectiveness of primary health care reforms in Greece towards achieving universal health coverage: a scoping review
Source: BMC Health Serv Res. 2021 Jul 1;21:628. doi: 10.1186/s12913-021-06678-9 (PMC8247133; doi:10.1186/s12913-021-06678-9)
Supplement: Supplementary file 1 — Additional file 1. [file 12913_2021_6678_MOESM1_ESM.docx]

**Supplementary material B**

**Included documents**

|  | **Title** | **Authors** | **Year** | **Method** | **Key findings** |
| --- | --- | --- | --- | --- | --- |
| 1 | A future for primary care for the Greek population | Groenewegen PP, Jurgutis A. | 2013 | Qualitative | Fragmented governance of primary health care and high OOP. High ratio of specialists against GPs and uneven distribution. Access, continuity, coordination and comprehensiveness need improvement, along with focus of medical training towards primary care. |
| 2 | ﻿Accessibility and use of health services by immigrants in Greece | ﻿Galanis, Petros Kaitelidou, Daphne Sourtzi, Panayota Siskou, Olga Konstantakopoulou, Olympia Economou, Charalambos | 2020 | Qualitative | Unequal access of migrants to health care services compared to local population. High OOP especially in medicines and long waiting times due to bureaucratic reasons or cultural barriers. |
| 3 | ﻿Assessment of users’ expectations, perceived quality and satisfaction with primary care in Greece | ﻿Raftopoulos, Vasilios | 2010 | Qualitative | Relatively high satisfaction from primary care services prior to 2010. Doctors more prestigious than nurses and rate of satisfaction heavily relies on their behavior and professionalism. Low satisfaction in average waiting time and consultation time. Expectations were greater prior to visit. |
| 4 | ﻿Barriers and facilitating factors in access to health services in Greece | ﻿Economou, Charalampos | 2015 | Qualitative | ﻿Significant problems in planning of healthcare personnel, development of medical technology and allocation of facilities. General oversupply of doctors coexists with medical understaffing of ESY. ﻿Persistent regional inequalities in the distribution of health resources, posing barriers to access especially for the population of remote areas and islands. High OOP and barriers to access due to bureaucracy. Low acceptability of the quality of services provided to patients and ﻿expression of racism against vulnerable groups. ﻿Routine monitoring of quality of care and clinical outcomes is insufficient. The development and introduction of medical records; clinical diagnostic and therapeutic protocols; diseases registries; and screening programmes should be of high priority. |
| 5 | Basic Principles, Positions and Proposals for the Development of Primary Health Care in Greece | ﻿Benos, Alexis Lionis, Christos Koutis, Antonis Antoniadou, Ioanna Panagiotopoulos, Takis Myloneros, Thanos | 2015 | Grey | Fragmented PHC services and unequal distribution. Supply induced demand based mostly on private sector. Recommendation for comprehensive primary care closer to the community (at the neighbourhood level), based on multidisciplinary teams. |
| 6 | ﻿Bridging knowledge to develop an action plan for integrated care for chronic diseases in Greece | ﻿Tsiachristas, Apostolos Lionis, Christos Yfantopoulos, John | 2016 | Qualitative | Chronic conditions are poorly managed and integrated care is still in embryonic stage. Lack of proper legal framework to support integrated chronic care models, limited leadership and advocacy, fragmentation of resources, misaligned incentives, lack of long-term investment and monitoring. Suggestions to emphasize prevention around the person, ensure sustainable finance. |
| 7 | ﻿Building primary care in a changing Europe – Greece | ﻿Lionis, Christos Tedeschi, Paolo Saridaki, Aristoula | 2015 | Qualitative | Rising prevalence of chronic diseases and lack of continuity in their management. Restricted access to primary care and little time for consultation, which affects also quality and chronic patients. Lack of pathways and gatekeeping and narrow spectrum of responsibilities for GPs and primary care professionals. |
| 8 | ﻿Can people afford to pay for health care? New evidence on financial protection in Europe | ﻿World Health Organization Regional Office for Europe | 2019 | Qualitative | High incidence of catastrophic health spending in the poorest households. Also, high levels of unmet need with income inequalities. The 2016 law improved access but has not addressed inequalities. ﻿People reliant on public facilities continue to face financial and non-financial barriers to access due to long waiting times and lack of staff, diagnostic equipment and supplies in public facilities |
| 9 | Citizen Preferences for Primary Health Care reform in Greece | Souliotis K, Tsimtsiou Z, Golna C, Nikolaidi S, Lionis C. | 2019 | Qualitative | ﻿The introduction of family physicians in the system was the most requested reform among PHC users. People preferred to visit private practices with an increasing trend to trust public services after the 2017 law. ﻿Older and female citizens were more likely to be in favor of this reform. Integrating the private sector in developing a comprehensive PHC system, enhancing existing public health services, and increasing public awareness of the advantages of PHC should be considered critical elements of a high-quality PHC system |
| 10 | Discussing issues of health promotion and research in the context of primary care during the ongoing austerity period: An exploratory analysis from two regions in Greece | Sbarouni V., Petelos E., Kamekis A., Tzagkarakis S.I., Symvoulakis E.K., Lionis C. | 2020 | Qualitative | Several gaps in terms of prevention and health promotion resources and activities. E-health, electronic record and telemedicine are underdeveloped. PHC is found to be inefficient with low quality. Monitoring and evaluation should be developed also based on evidence through research in PHC. |
| 11 | Evaluation of the Operation of the Local Health Care Teams (ΤΟΜΥ): Executive Summary of Conclusions and Recommendations | NSRF Executive Agency, Ministry of Health | 2020 | Grey | Operational assessment of the TOMYs conducted for the Ministry of Health under the framework of the NSRF funding. Main conclusions include positive results in terms of staff and patient satisfaction and expansion of community services, but also weaknesses in the completion of the roll-out due to lack of human resources and premises and underdeveloped integration. |
| 12 | Exploring barriers to primary care for migrants in Greece in times of austerity: Perspectives of service providers | Papadakaki M., Lionis C., Saridaki A., Dowrick C., De Brún T., O’Reilly-De Brún M., O’Donnell C.A., Burns N., Van Weel-Baumgarten E., Van Den Muijsenbergh M., Spiegel W., MacFarlane A. | 2017 | Qualitative | Discriminatory attitudes and other provider and system-related barriers are evident in the provision of primary healthcare to migrants in Greece. Providers feel unable to fulfil their role efficiently under limited system support and contribution to decision making. Training and guidelines promoting cultural competence are necessary in the Greek primary healthcare. |
| 13 | Exploring health care reform in a changing Europe: Lessons from Greece | Kousoulis A.A., Angelopoulou K.-E., Lionis C. | 2013 | Qualitative | ﻿The economic crisis led to a shift towards PHC. Apart from healthcare, Greece should focus on general changes, primarily decentralization and education. |
| 14 | ﻿Greece: Country Health Profile 2019 | ﻿OECD, European Observatory on Health Systems and Policies | 2019 | Qualitative | Greece’s health system has undergone a major transformation, slowly moving towards a more modern, efficient and sustainable system. After an initial focus on efficiency enhancing structural reforms and cost reductions, more recent efforts have also focused on introducing and strengthening mechanisms to achieve better outcomes. There is now comprehensive health insurance coverage for all residents and Greece is working on establishing a functioning primary care system. ﻿The roll-out of primary care since 2017 has proceeded at a relatively good pace, with just over half (127) of the planned primary care units operating in the summer of 2019. |
| 15 | ﻿Greece: Health System Review | ﻿Economou, Charalampos Kaitelidou, Daphne Karanikolos, Marina Maresso, Anna | 2017 | Qualitative | ﻿A GP-based comprehensive, integrated primary health care system with gatekeeping functions is lacking, particularly in urban areas. Existing primary care is neither well developed nor well organized, while health services are unilaterally oriented towards acute health problems, rarely engaging in health promotion or disease prevention. Furthermore, integration of health and social services and the development of long-term ﻿care have not been explicitly included in the reform agenda. The newly announced primary care reforms, which began implementation in 2017, are an evidence-based response to these challenges. |
| 16 | ﻿Health Literacy in the Everyday Lives of Older Adults in Greece, Hungary, and the Netherlands | ﻿De Wit, Liesbeth Karnaki, Pania Dalma, Archontoula Csizmadia, Peter Salter, Charlotte de Winter, Andrea Meijering, Louise | 2020 | Qualitative | Poor health literacy is associated with low socioeconomic status. Patients believe that their input is necessary for correct diagnosis but older people never questioned the doctor’s decision. Insufficient time for consultation that does not give the space for gaining trust and empower patients. Private health services are thought to be of better quality and preferred even though more expensive. |
| 17 | ﻿Human Resources for Health in Greece: Current status and the way forward | ﻿Kaitelidou, Daphne Economou, Charalambos Siskou, Olga Konstantakopoulou, Olympia Galanis, Petros Myloneros, Thanos Domente, Silviu | 2018 | Qualitative | ﻿Greece holds the highest proportion of doctors (6.6/1,000 inhabitants) among the EU countries, with an increasing trend over the years, but ﻿the last position among the EU countries for nursing staff, with a proportion of nursing staff of 3.3 per 1,000 inhabitants. There is ﻿shortage in GPs as their ratio in 2016 was among the lowest in EU-15 with only 0.4/1000 inhabitants. ﻿Lack of proper planning, national health workforce database and registries, increased mobility status (brain drain), payments not related to performance, curricula need to address modern needs and CPD is necessary. |
| 18 | ﻿Inequalities Between Migrants and Non-Migrants in Accessing and Using Health Services in Greece During an Era of Economic Hardship | ﻿Kaitelidou, Daphne Galanis, Petros Economou, Charalambos Mladovsky, Philipa Siskou, Olga Sourtzi, Panayota | 2020 | Mixed | ﻿Migrants, people suffering from a chronic disease, those without health insurance, and patients who assessed their health status as not at all good/a little good/moderate were statistically more likely to report unmet needs in getting their medication. Greece at the time, despite administrative delays and barriers, provided full coverage to the uninsured, asylum seekers, and migrants, even many groups of undocumented migrants. |
| 19 | Informing primary care reform in Greece: patient expectations and experiences (the QUALICOPC study) | Lionis C., Papadakis S., Tatsi C., Bertsias A., Duijker G., Mekouris P.-B., Boerma W., Schäfer W. | 2017 | Qualitative | Most patients surveyed reported good rates of satisfaction with their experience in the health care system. However, several areas for improvement were also identified, including issues of accessibility, patient-centredness, patient involvement and continuity of care. ﻿The focus on urgent care issues and lack of comprehensive approach to family or community-based health care delivery is highlighted. ﻿A positive finding of the survey is that most of the patients (especially patients with chronic disease, who visit primary care unit more regularly) surveyed report that their health needs are being met. |
| 20 | Institutional and Organizational Efforts to Establish Primary Health Care in Greece | Platis, Charalampos; Kyritsi, Nikoletta; | 2019 | Qualitative | ﻿The 2017 reform fulfilled the fundamental principles of PHC. Planning of long-term funding is incomplete and ﻿there is poor progress to the implementation timetable of the reform is recorded due to stuffing difficulties. Recommendations: Incentives for staffing are needed; recording, monitoring and audit of health indicators;  on-the-job training of internists who will have a family doctor role in the new system; recording and evaluating long-term structures, processes and results so that they can reasonably determine what direction the system should take; use of information and communication technologies to bridge access disparities in remote areas. |
| 21 | ﻿Integrated health care services as a current challenge for primary health care: reflections from Crete, Greece | ﻿Lionis, Christos; Anastasaki, Marilena; | 2019 | Qualitative | ﻿Lack of involvement of patients, families, and communities in health care. Need for ﻿training of stakeholders to define and promote integrated care. ﻿Lack of advocacy and care coordination. |
| 22 | Integrated people-centred primary health care in Greece: Unravelling Ariadne's thread | Lionis C., Symvoulakis E.K., Markaki A., Petelos E., Papadakis S., Sifaki-Pistolla D., Papadakakis M., Souliotis K., Tziraki C. | 2019 | Qualitative | ﻿PHC in Greece is still far from embracing and utilizing to the full extent the key concepts in consensus documents and WHO declarations. ﻿Fragmentation of services and lack of consistent direction in the management of chronic diseases and major public health risks are on-going handicaps. Emphasis should be given on integration of PHC and public health, development of competencies, interdisciplinary education and training, workforce planning and chronic care management. |
| 23 | Investigating unmet health needs in primary health care services in a representative sample of the Greek population | Pappa E., Kontodimopoulos N., Papadopoulos A., Tountas Y., Niakas D. | 2013 | Qualitative | ﻿The most frequently self-reported reasons for unmet needs were cost and lack of time. Youth, parenthood, physician consultations, and poor mental health increased the likelihood of unmet needs. Women were less likely to report accessibility and availability than acceptability barriers. Unmet needs were related both to individuals and health system characteristics and important educational differences were raised. Attention should be paid by health care policy makers and focused policy actions should be undertaken in order to facilitate appropriate help-seeking and service use. ﻿Physician consultations increase the likelihood of reporting unmet needs by about two times |
| 24 | Law 3918: Structural Changes In The Health System And Other Provisions. | Hellenic Republic | 2011 | Parliamentary Law |  |
| 25 | Law 4238: Primary National Health Network (PEDY), change of EOPYY's purpose and other provisions. | Hellenic Republic | 2014 | Parliamentary Law |  |
| 26 | Law 4368: Measures to acccellerate government work and other provisions. | Hellenic Republic | 2016 | Parliamentary Law |  |
| 27 | Law 4486: Reform of Primary Health Care, urgent regulations of the Ministry of Health and other provisions. | Hellenic Republic | 2017 | Parliamentary Law |  |
| 28 | Measuring the efficiency of the Greek rural primary health care using a restricted DEA model; the case of southern and western Greece | Oikonomou N, Tountas Y, Mariolis A, Souliotis K, Athanasakis K, Kyriopoulos J. | 2016 | Qualitative | ﻿A notable number of facilities located in rural areas of Southern and Western Greece were found under-performing to some extent, with considerable variation between units. This result can be attributed to both structural and functional insufficiencies of the primary care system. Efficiency improvement in rural primary care provision in Greece is feasible and can be accomplished through the development of a more integrated and comprehensive primary health care system and through better management of re- sources, accompanied by radical reforms and updated organizational policies. |
| 29 | ﻿Monitoring and documenting systemic and health effects of health reforms in Greece | ﻿Economou, Charalampos Panteli, Dimitra | 2019 | Qualitative | ﻿The new PHC system embodies the fundamental principles of WHO and is expected to result in better access to quality health care and more rational and efficient use of existing services and resources by reducing unnecessary hospital admittance through well-organized referral processes. Nevertheless, a number of challenges emerge for policy-makers to address: (i) teamwork, health- promotion activities, community empowerment and prevention programmes have traditionally existed in the margins of care in Greece; (ii) health professionals lack of experience of teamwork for outward activities; (iii) lack of well-defined procedures and coordination of processes in delivery of care in different settings; (iv) doctors’ unwillingness to work in TOMYs; (v) difficulty in organizing and maintaining impetus for short-term training programmes on teamwork and work processes in primary care; (vi) electronic medical record is not yet fully operational; and (vii) some clinical guidelines have been developed but there is still the challenge of training personnel to incorporate their content into everyday practice. Possible solutions to some of these issues include: (a) building on work of local pioneers to further foster interdisciplinary teamwork; (b) ensuring integration of health promotion and disease prevention interventions at individual and community outreach level; (c) ensuring effective coordination internally (within TOMYs) and externally based on collaborative rather than authoritative formats; (d) enhancing communication using cascading measures from national, to regional and municipal levels to help combat skepticism among the general public, local ﻿communities and health professionals; and (e) redefining salary levels and employment contracts for TOMY staff and EOPYY-contracted practitioners to prevent differentiated incentives. |
| 30 | Nationwide epidemiological study of knowledge, attitudes, and practices study of Greek general practitioners related to screening | Dresios C., Rachiotis G., Symvoulakis E.K., Rousou X., Papagiannis D., Mouchtouri V., Hadjichristodoulou C. | 2019 | Qualitative | GPs agreed on the key role of population-based screening in improving patient care and most reported that their performance would be improved with the adoption of computer-based support systems in clinical practice. GPs, older than 50 years of age, those who those practicing for more than 15 years and GPs working in private sector, were less likely to comply with screening recommendations. Latent class analysis revealed that male physicians and those working in public sector were more likely to comply with USPSTF recommendations. There is need of educational intervention programs for GPs in order to promote the implementation of national evidence-based screening recommendation statements in clinical practice. |
| 31 | Operational integration in primary health care: Patient encounters and workflows | Sifaki-Pistolla D., Chatzea V.-E., Markaki A., Kritikos K., Petelos E., Lionis C. | 2017 | Mixed | Average or below average levels of patient-level integration within PHC units, with variations based on type of PHC services rendered. Indications for a fragmented and ineffective healthcare system in need of reform were evident, particularly when assessing the existing patient care pathways. The web-based evaluation tool, along with the proposed patient-level operational integration model, could become the core elements for an overall sound and cost-effective primary healthcare system, a system where professionals, along with patients, are motivated and empowered to work collectively towards integrated patient-centred care. |
| 32 | Operational integration of primary health care units in Greece: Mapping of the current status | Karagianni, M; Sifaki-Pistolla, D; Chatzea, VE; Trigoni, M; Koutis, A; Petelos, E; Lionis, C; | 2017 | Qualitative | The majority of the PHC units ranged between basic to partial integration, while a small percentage (5%) were rated as poorly integrated. Continuity was reported to have a higher degree of integration, whereas the dimensions that were identified as requiring significant improvement were “Economic Conditions of the PHC system” and the “Service Quality of PHC”. In the majority of the units, the rating regarding economic conditions was evaluated below the basic level. A high degree of heterogeneity was identified across PHC services in Greece. The implementation of targeted actions could affect improvement in the quality of services provided and in the degree of operational integration, thus enhancing their effectiveness. |
| 33 | Patients’ Satisfaction with the National Primary Health Care Net in Greece | Frengidou, Elisavet; Galanis, Petros; | 2020 | Qualitative | ﻿The level of overall satisfaction among participants was relatively low (48.6%). Increased age, waiting time satisfaction, medical satisfaction score and intention of recommendation of the services provided to others were related with increased overall satisfaction. The most important findings were reduced frequency of use, users’ preference to private doctors, short duration of medical visit and a lack of medical specialties. Inadequate interconnection among primary or secondary healthcare level was also noted as well as high rates of preference concerning medical referral to specialists and hospitals by family doctors. |
| 34 | Perceptions of primary care professionals on quality of services in rural Greece: a qualitative study | Sbarouni V, Tsimtsiou Z, Symvoulakis E, Kamekis A, Petelos E, Saridaki A, Papadakis N, Lionis C. | 2012 | Qualitative | ﻿The main identified barriers to providing high-quality PHC services were: PHC service shortages in workforce and equipment; inadequate GP and paramedic training; the absence of position/job descriptions or duty statements for GPs and other PHC personnel; and limited public awareness about the role of GPs. Suggestions for remodeling the current PHC system included: the introduction of new technologies; GP empowerment; leadership reforms; and mechanisms for evaluating of the quality of services. Finally, areas of concern regarding future development and utilization of private PHC infrastructure and services were highlighted. |
| 35 | Primary care doctors’ assessment of and preferences on their remuneration: Evidence from Greek public sector | Karakolias S., Kastanioti C., Theodorou M., Polyzos N. | 2017 | Qualitative | PHC doctors are extremely dissatisfied with the current reimbursement method, however they do not reach a consensus to change it. Only GPs of higher medical grades look ready to replace their ostensibly fixed income with per capita payments and/or FFS. This conceals that probably dissatisfaction relies on the amount of remuneration instead of the type of payment itself. However, radical changes in the remuneration of GPs, who have already expressed their preference on capitation through their professional associations, and adjustments to the remuneration of all medical specialties by introducing incentives (pay-for- performance), can lead to improved quality and access. In addition, the competent authorities have a great opportunity to reform the organizational structure of PHC using adjustments to remuneration and employment conditions as an intermediate objective |
| 36 | Psychiatrists role in primary health care in Greece: Findings from a quantitative study | Souliotis K., Agapidaki E., Tzavara C., Economou M. | 2017 | Mixed | ﻿Primary care practitioners cited lack of collaboration and communication with psychiatrists as key barriers for the effective mental health management in primary care. ﻿Psychiatrists are receptive to participate in primary care although important barriers were reported. Also, ﻿primary care practitioners were more confident dealing with mental health problems than psychiatrists felt in treating physical conditions. OOP for mental health services remain high notwithstanding the financial crisis and the integration of mental health into primary care should be a key priority. |
| 37 | ﻿Re-profiling emergency medical services in Greece | ﻿World Health Organization Regional Office for Europe | 2017 | Qualitative | ﻿Viable primary care that manages chronic conditions and is responsive to acute health needs is a precondition for re-profiling emergency medical services. ﻿There is also a need to define a scope of practice of primary care providers that includes roles and responsibilities on health promotion, disease prevention while increasing their responsive capacity in diagnosing, treating and managing people with noncommunicable diseases. ﻿The alignment of providers’ payment mechanisms for primary care teams should complement these efforts. ﻿In order to effectively assume the first line of responsibility for managing acute care needs, primary care will need to be made available 24/7. |
| 38 | Reinventing primary health care in the Greece of austerity: the role of health-care workers | Simou E, Karamagioli E, Roumeliotou A. | 2015 | Quantitative | PHC absorbs a very limited part of the national health system’s workforce. Important inequalities in the numerical and geographical allocation of the PHC health workforce specialties across the country in favor of the medical profession and to the detriment of rural areas and the islands were identified, raising concerns about the policymakers’ ability to meet the emerging needs of the population, as the retrospective study of the health-care workforce, since 2010, reveals that the numerical and per type allocations remained almost unchanged. These results were in line with previous studies showcasing the lack of holistic approach for PHC questioning the restrictive spending policy (ie, salary and benefit cuts for the health-care professionals, important discharges and nonrenewal of the personnel) adopted in the public health-care sector. |
| 39 | Rural primary care in Greece: Working under limited resources | Oikonomidou E., Anastasiou F., Dervas D., Patri F., Karaklidis D., Moustakas P., Andreadou N., Mantzanas E., Merkouris B. | 2010 | Qualitative | ﻿Shortages in personnel (GPs and nurses) and equipment within the primary care practices both in the mainland and the small islands. The lack of community nurses is another drawback of the Greek rural primary health-care system. Lack of medical record documentation in the majority of RS, raising issues of the continuity of health care as well as safety concerns. The number of GPs keeping medical records was four times higher than that of non-specialized doctors. |
| 40 | ﻿Solidarity outpatient clinics in Greece: a survey of a massive social movement | ﻿Evlampidou, Iro Kogevinas, Manolis | 2019 | Qualitative | In response to the barriers created by the, solidarity outpatient clinics provided preventive, chronic and emergency healthcare to, mostly, uninsured people., substituting thus PHC. That indicates a clear lack of access to care. ﻿The largest clinics examined more than 500 uninsured or partly insured patients per month. Clinics covered a wide range of clinical and preventive services. Funding, availability of drugs, vaccines, medical material and their legal status were the main problems identified. The solidarity movement involved thousands of health professionals covering essential population needs. |
| 41 | The integration of mental health services into primary health care in Greece. | Peritogiannis, V; Lixouriotis, C; Mavreas, V; | 2014 | Qualitative | ﻿It is generally accepted that common mental disorders are highly prevalent in the clinical populations of the primary care system, but that they tend to be under-recognized and under-treated by primary care physicians. In addition, patients with chronic psychosis often present physical morbidity requiring primary care. Contemporary models of integrated care include the co-location of psychiatric or other trained mental health staff in the primary care setting, liaison of services and the establishment of community mental health teams, which in the rural areas of Greece operate as mobile mental health units (MMHUs). The MMHUs in Greece provide multi-level care at low cost, and therefore constitute the optimal way of mental health integration into primary care in the era of economic cri- sis, when needs are increased and resources are limited. The Greek government is advised to invest in such services, by ensuring adequate funding. |
| 42 | The newly established unified healthcare fund (EOPYY): current situation and proposed structural changes, towards an upgraded model of primary health care, in Greece | Karakolias, Stefanos E; Polyzos, Nikolaos M; | 2014 | Qualitative | ﻿﻿PHC provision is fragmented leading patients to more expensive hospital care. Family physicians are a small portion of total physicians which, in combination with the free choice policy, results in non-gatekeeping despite growing co-payments. This necessitates the creation of a PHC network between EOPYY’s and NHS’s units and contracted professionals. This first evaluation has also revealed an irrational use of consolidated resources, which we propose to normalize through a new global budget system. EOPYY’s establishment reform in 2011 may turn into a disaster if a PHC orientation is not adopted whereby GPs and family pediatricians control patients flow in the NHS. |
| 43 | Use of primary health care services in Southern Greece during a period of economic crisis. | Patelarou, AE; Kleisiaris, CF; Androulakis, E; Tsirakos, DK; Kritsotakis, G; Konstantinidis, TΙ; Androulaki, Z; | 2016 | Quantitative | ﻿Respiratory, musculoskeletal, and digestive symptoms were identified as the most frequent reasons for emergency visits to PHCCs in rural Crete. Neuropsychiatric symptoms were observed to have increased since the onset of the economic crisis. This study revealed variations in the frequency of the presenting symptoms according to gender, age, place of residence, year and season of the PHCC visit. The study data justify the urgent need for the development and adoption of electronic health records in PHCCs and confirm that the re-orientation of the Greek health system to focus on PHC and public health is now more essential than ever. |
| 44 | ﻿Using patient experience measures to evaluate the quality of medical and nursing care in the newly established Primary Health Care units (TOMYs), in Greece | ﻿Konstantakopoulou, Olympia Kaitelidou, Daphne Galanis, Petros Siskou, Olga Economou, Charalambos | 2019 | Qualitative | Users of the TOMYs’ services reported positive experiences at all factors evaluated. All mean scores indicated positive levels of patient experience (﻿Accessibility, Continuity/coordination of care, Comprehensiveness of care, Quality of medical care, Facility, Quality of nursing care). The quality of medical and nursing care had the highest mean scores. Positive findings must be further pursued so as for these units to uphold to their stated objective and mission. |

**Supplementary material C**

**Critical appraisal**

***MMAT Qualitative***

| **Title** | **Authors** | **Year** | **Method** | **Are there clear research questions?** | **Do the collected data allow to address the research questions?** | **Is the qualitative approach appropriate to answer the research question?** | **Are the qualitative data collection methods adequate to address the research question?** | **Are the findings adequately derived from the data?** | **Is the interpretation of results sufficiently substantiated by data?** | **Is there coherence between qualitative data sources, collection, analysis and interpretation?** | **SCORE** |
| --- | --- | --- | --- | --- | --- | --- | --- | --- | --- | --- | --- |
| A future for primary care for the Greek population | Groenewegen PP, Jurgutis A. | 2013 | Qualitative | Yes | Yes | Yes | Yes | Yes | Yes | Yes | 100% |
| ﻿Accessibility and use of health services by immigrants in Greece | ﻿Galanis, Petros Kaitelidou, Daphne Sourtzi, Panayota Siskou, Olga Konstantakopoulou, Olympia Economou, Charalambos | 2020 | Qualitative | Yes | Yes | Yes | Yes | Yes | Yes | Yes | 100% |
| ﻿Assessment of users’ expectations, perceived quality and satisfaction with primary care in Greece | ﻿Raftopoulos, Vasilios | 2010 | Qualitative | Yes | Yes | Yes | Yes | Yes | Yes | Yes | 100% |
| ﻿Barriers and facilitating factors in access to health services in Greece | ﻿Economou, Charalampos | 2015 | Qualitative | Yes | Yes | Yes | Yes | Yes | Yes | Yes | 100% |
| ﻿Bridging knowledge to develop an action plan for integrated care for chronic diseases in Greece | ﻿Tsiachristas, Apostolos Lionis, Christos Yfantopoulos, John | 2016 | Qualitative | Yes | Yes | Yes | Yes | Yes | Yes | Yes | 100% |
| ﻿Building primary care in a changing Europe – Greece | ﻿Lionis, Christos Tedeschi, Paolo Saridaki, Aristoula | 2015 | Qualitative | Yes | Yes | Yes | Yes | Yes | Yes | Yes | 100% |
| ﻿Can people afford to pay for health care? New evidence on financial protection in Europe | ﻿World Health Organization Regional Office for Europe | 2019 | Qualitative | Yes | Yes | Yes | Yes | Yes | Yes | Yes | 100% |
| Citizen Preferences for Primary Health Care reform in Greece | Souliotis K, Tsimtsiou Z, Golna C, Nikolaidi S, Lionis C. | 2019 | Qualitative | Yes | Yes | Yes | Yes | Yes | Yes | Yes | 100% |
| Discussing issues of health promotion and research in the context of primary care during the ongoing austerity period: An exploratory analysis from two regions in Greece | Sbarouni V., Petelos E., Kamekis A., Tzagkarakis S.I., Symvoulakis E.K., Lionis C. | 2020 | Qualitative | Yes | Yes | Yes | N/A | Yes | Yes | Yes | 80% |
| Exploring barriers to primary care for migrants in Greece in times of austerity: Perspectives of service providers | Papadakaki M., Lionis C., Saridaki A., Dowrick C., De Brún T., O’Reilly-De Brún M., O’Donnell C.A., Burns N., Van Weel-Baumgarten E., Van Den Muijsenbergh M., Spiegel W., MacFarlane A. | 2017 | Qualitative | Yes | Yes | Yes | Yes | Yes | Yes | Yes | 100% |
| Exploring health care reform in a changing Europe: Lessons from Greece | Kousoulis A.A., Angelopoulou K.-E., Lionis C. | 2013 | Qualitative | Yes | Yes | Yes | Yes | Yes | Yes | Yes | 100% |
| ﻿Greece: Country Health Profile 2019 | ﻿OECD, European Observatory on Health Systems and Policies | 2019 | Qualitative | Yes | Yes | Yes | Yes | Yes | Yes | Yes | 100% |
| ﻿Greece: Health System Review | ﻿Economou, Charalampos Kaitelidou, Daphne Karanikolos, Marina Maresso, Anna | 2017 | Qualitative | Yes | Yes | Yes | Yes | Yes | Yes | Yes | 100% |
| ﻿Health Literacy in the Everyday Lives of Older Adults in Greece, Hungary, and the Netherlands | ﻿De Wit, Liesbeth Karnaki, Pania Dalma, Archontoula Csizmadia, Peter Salter, Charlotte de Winter, Andrea Meijering, Louise | 2020 | Qualitative | Yes | Yes | Yes | No | Yes | Yes | Yes | 100% |
| ﻿Human Resources for Health in Greece: Current status and the way forward | ﻿Kaitelidou, Daphne Economou, Charalambos Siskou, Olga Konstantakopoulou, Olympia Galanis, Petros Myloneros, Thanos Domente, Silviu | 2018 | Qualitative | Yes | Yes | Yes | Yes | Yes | Yes | Yes | 100% |
| Informing primary care reform in Greece: patient expectations and experiences (the QUALICOPC study) | Lionis C., Papadakis S., Tatsi C., Bertsias A., Duijker G., Mekouris P.-B., Boerma W., Schäfer W. | 2017 | Qualitative | Yes | Yes | Yes | Yes | Yes | Yes | Yes | 100% |
| Institutional and Organizational Efforts to Establish Primary Health Care in Greece | Platis, Charalampos; Kyritsi, Nikoletta; | 2019 | Qualitative | Yes | Yes | Yes | Yes | No | No | Yes | 60% |
| ﻿Integrated health care services as a current challenge for primary health care: reflections from Crete, Greece | ﻿Lionis, Christos; Anastasaki, Marilena; | 2019 | Qualitative | Yes | Yes | Yes | Yes | Yes | Yes | Yes | 100% |
| Integrated people-centred primary health care in Greece: Unravelling Ariadne's thread | Lionis C., Symvoulakis E.K., Markaki A., Petelos E., Papadakis S., Sifaki-Pistolla D., Papadakakis M., Souliotis K., Tziraki C. | 2019 | Qualitative | Yes | Yes | Yes | Yes | Yes | Yes | Yes | 100% |
| Investigating unmet health needs in primary health care services in a representative sample of the Greek population | Pappa E., Kontodimopoulos N., Papadopoulos A., Tountas Y., Niakas D. | 2013 | Qualitative | Yes | Yes | Yes | Yes | Yes | Yes | Yes | 100% |
| Measuring the efficiency of the Greek rural primary health care using a restricted DEA model; the case of southern and western Greece | Oikonomou N, Tountas Y, Mariolis A, Souliotis K, Athanasakis K, Kyriopoulos J. | 2016 | Qualitative | Yes | Yes | Yes | Yes | Yes | Yes | Yes | 100% |
| ﻿Monitoring and documenting systemic and health effects of health reforms in Greece | ﻿Economou, Charalampos Panteli, Dimitra | 2019 | Qualitative | Yes | Yes | Yes | Yes | Yes | Yes | Yes | 100% |
| Nationwide epidemiological study of knowledge, attitudes, and practices study of Greek general practitioners related to screening | Dresios C., Rachiotis G., Symvoulakis E.K., Rousou X., Papagiannis D., Mouchtouri V., Hadjichristodoulou C. | 2019 | Qualitative | Yes | Yes | Yes | Yes | Yes | Yes | Yes | 100% |
| Operational integration of primary health care units in Greece: Mapping of the current status | Karagianni, M; Sifaki-Pistolla, D; Chatzea, VE; Trigoni, M; Koutis, A; Petelos, E; Lionis, C; | 2017 | Qualitative | Yes | Yes | Yes | Yes | Yes | Yes | Yes | 100% |
| Patients’ Satisfaction with the National Primary Health Care Net in Greece | Frengidou, Elisavet; Galanis, Petros; | 2020 | Qualitative | Yes | Yes | Yes | Yes | Yes | Yes | Yes | 100% |
| Perceptions of primary care professionals on quality of services in rural Greece: a qualitative study | Sbarouni V, Tsimtsiou Z, Symvoulakis E, Kamekis A, Petelos E, Saridaki A, Papadakis N, Lionis C. | 2012 | Qualitative | Yes | Yes | Yes | Yes | Yes | Yes | Yes | 100% |
| Primary care doctors’ assessment of and preferences on their remuneration: Evidence from Greek public sector | Karakolias S., Kastanioti C., Theodorou M., Polyzos N. | 2017 | Qualitative | Yes | Yes | Yes | Yes | Yes | Yes | Yes | 100% |
| ﻿Re-profiling emergency medical services in Greece | ﻿World Health Organization Regional Office for Europe | 2017 | Qualitative | Yes | Yes | Yes | Yes | Yes | No | Yes | 80% |
| Rural primary care in Greece: Working under limited resources | Oikonomidou E., Anastasiou F., Dervas D., Patri F., Karaklidis D., Moustakas P., Andreadou N., Mantzanas E., Merkouris B. | 2010 | Qualitative | Yes | Yes | Yes | Yes | Yes | Yes | Yes | 100% |
| ﻿Solidarity outpatient clinics in Greece: a survey of a massive social movement | ﻿Evlampidou, Iro Kogevinas, Manolis | 2019 | Qualitative | Yes | Yes | Yes | No | Yes | Yes | Yes | 80% |
| The integration of mental health services into primary health care in Greece. | Peritogiannis, V; Lixouriotis, C; Mavreas, V; | 2014 | Qualitative | Yes | Yes | Yes | Yes | No | Yes | Yes | 80% |
| The newly established unified healthcare fund (EOPYY): current situation and proposed structural changes, towards an upgraded model of primary health care, in Greece | Karakolias, Stefanos E; Polyzos, Nikolaos M; | 2014 | Qualitative | Yes | Yes | Yes | Yes | Yes | Yes | Yes | 100% |
| ﻿Using patient experience measures to evaluate the quality of medical and nursing care in the newly established Primary Health Care units (TOMYs), in Greece | ﻿Konstantakopoulou, Olympia Kaitelidou, Daphne Galanis, Petros Siskou, Olga Economou, Charalambos | 2019 | Qualitative | Yes | Yes | Yes | Yes | Yes | Yes | Yes | 100% |

###

**Supplementary material D**

**Critical appraisal**

***MMAT Quantitative***

| **Title** | **Authors** | **Year** | **Method** | **Are there clear research questions?** | **Do the collected data allow to address the research questions?** | **Is the sampling strategy relevant to address the research question?** | **Is the sample representative of the target population?** | **Are the measurements appropriate?** | **Is the risk of nonresponse bias low?** | **Is the statistical analysis appropriate to answer the research question?** | **Score** |
| --- | --- | --- | --- | --- | --- | --- | --- | --- | --- | --- | --- |
| Reinventing primary health care in the Greece of austerity: the role of health-care workers | Simou E, Karamagioli E, Roumeliotou A. | 2015 | Quantitative | Yes | Yes | Yes | Yes | Yes | Yes | Yes | 100% |
| Use of primary health care services in Southern Greece during a period of economic crisis. | Patelarou, AE; Kleisiaris, CF; Androulakis, E; Tsirakos, DK; Kritsotakis, G; Konstantinidis, TΙ; Androulaki, Z; | 2016 | Quantitative | Yes | Yes | Yes | Yes | Yes | Yes | Yes | 100% |

**Supplementary material FE**

**Critical appraisal**

***MMAT Mixed Methods***

| **Title** | **Authors** | **Year** | **Method** | **Are there clear research questions?** | **Do the collected data allow to address the research questions?** | **Is the sampling strategy relevant to address the research question?** | **Is the sample representative of the target population?** | **Are the measurements appropriate?** | **Is the risk of nonresponse bias low?** | **Is the statistical analysis appropriate to answer the research question?** | **SCORE** |
| --- | --- | --- | --- | --- | --- | --- | --- | --- | --- | --- | --- |
| ﻿Inequalities Between Migrants and Non-Migrants in Accessing and Using Health Services in Greece During an Era of Economic Hardship | ﻿Kaitelidou, Daphne Galanis, Petros Economou, Charalambos Mladovsky, Philipa Siskou, Olga Sourtzi, Panayota | 2020 | Mixed | Yes | Yes | Yes | Yes | Yes | Yes | Yes | 100% |
| Operational integration in primary health care: Patient encounters and workflows | Sifaki-Pistolla D., Chatzea V.-E., Markaki A., Kritikos K., Petelos E., Lionis C. | 2017 | Mixed | Yes | Yes | Yes | Yes | Yes | No | Yes | 80% |
| Psychiatrists role in primary health care in Greece: Findings from a quantitative study | Souliotis K., Agapidaki E., Tzavara C., Economou M. | 2017 | Mixed | Yes | Yes | Yes | Yes | Yes | Yes | Yes | 100% |

**Supplementary material F**

**Critical appraisal**

***AACODS***

| **Title** | **Basic Principles, Positions and Proposals for the Development of Primary Health Care in Greece** |  |  |  |  |
| --- | --- | --- | --- | --- | --- |
| Author(s) | Benos, Alexis / Lionis, Christos / Koutis, Antonis / Antoniadou, Ioanna / Panagiotopoulos, Takis / Myloneros, Thanos |  |  |  |  |
| **Year** | **2015** |  |  |  |  |
|  | | | | | |
| **Authority** | Associated with a reputable organization? | Yes |  |  |  |
|  | Professional qualifications or considerable experience? | Yes |  |  |  |
|  | Produced/published other work (grey/black) in the field? | Yes |  |  |  |
|  | Recognized expert, identified in other sources? | Yes |  |  |  |
|  | Cited by others? | Yes |  |  |  |
|  | Higher degree student under “expert” supervision? |  | No |  |  |
|  | Does the item have a detailed reference list or bibliography? | Yes |  |  |  |
| **Accuracy** | Does the item have a clearly stated aim or brief? | Yes |  |  |  |
|  | If so, is this met? | Yes |  |  |  |
|  | Does it have a stated methodology? |  | No |  |  |
|  | If so, is it adhered to? |  |  | N/A |  |
|  | Has it been peer-reviewed? |  | No |  |  |
|  | Has it been edited by a reputable authority? |  | No |  |  |
|  | Supported by authoritative, documented references or credible sources? | Yes |  |  |  |
|  | Is it representative of work in the field? | Yes |  |  |  |
|  | If No, is it a valid counterbalance? |  |  | N/A |  |
|  | Is any data collection explicit and appropriate for the research? | Yes |  |  |  |
|  | If item is secondary material (e.g. a policy brief of a technical report) refer to the original. | Yes |  |  |  |
|  | Is it an accurate, unbiased interpretation or analysis? |  |  |  | Can’t tell |
| **Coverage** | Are any limits clearly stated? |  | No |  |  |
| **Objectivity** | Opinion, expert or otherwise, is still opinion: is the author’s standpoint clear? | Yes |  |  |  |
|  | Does the work seem to be balanced in presentation? | Yes |  |  |  |
| **Date** | Does the item have a clearly stated date related to content? No easily discernible date is a strong concern. | Yes |  |  |  |
|  | If no date is given, but can be closely ascertained, is there a valid reason for its absence? |  |  | N/A |  |
|  | Check the bibliography: have key contemporary material been included? | Yes |  |  |  |
| **Significance** | Is the item meaningful? (feasibility, utility and relevance) | Yes |  |  |  |
|  | Does it add context? | Yes |  |  |  |
|  | Does it enrich or add something unique to the research? | Yes |  |  |  |
|  | Does it strengthen or refute a current position? | Yes |  |  |  |
|  | Would the research area be lesser without it? |  | No |  |  |
|  | Is it integral, representative, typical? | Yes |  |  |  |
|  | Does it have impact? (in the sense of influencing the work or behavior of others) | Yes |  |  |  |

| **Title** | **Evaluation of the Operation of the Local Health Care Teams (ΤΟΜΥ)** |  |  |  |  |
| --- | --- | --- | --- | --- | --- |
| Author(s) | NSRF Executive Agency, Ministry of Health |  |  |  |  |
| **Year** | **2020** |  |  |  |  |
|  | | | | | |
| **Authority** | Associated with a reputable organization? | Yes |  |  |  |
|  | Professional qualifications or considerable experience? | Yes |  |  |  |
|  | Produced/published other work (grey/black) in the field? | Yes |  |  |  |
|  | Recognized expert, identified in other sources? | Yes |  |  |  |
|  | Cited by others? |  |  |  | Can’t tell |
|  | Higher degree student under “expert” supervision? |  | No |  |  |
|  | Does the item have a detailed reference list or bibliography? |  | No |  |  |
| **Accuracy** | Does the item have a clearly stated aim or brief? | Yes |  |  |  |
|  | If so, is this met? | Yes |  |  |  |
|  | Does it have a stated methodology? | Yes |  |  |  |
|  | If so, is it adhered to? | Yes |  |  |  |
|  | Has it been peer-reviewed? |  | No |  |  |
|  | Has it been edited by a reputable authority? |  | No |  |  |
|  | Supported by authoritative, documented references or credible sources? |  | No |  |  |
|  | Is it representative of work in the field? | Yes |  |  |  |
|  | If No, is it a valid counterbalance? |  |  | N/A |  |
|  | Is any data collection explicit and appropriate for the research? | Yes |  |  |  |
|  | If item is secondary material (e.g. a policy brief of a technical report) refer to the original. | Yes |  |  |  |
|  | Is it an accurate, unbiased interpretation or analysis? | Yes |  |  |  |
| **Coverage** | Are any limits clearly stated? |  | No |  |  |
| **Objectivity** | Opinion, expert or otherwise, is still opinion: is the author’s standpoint clear? | Yes |  |  |  |
|  | Does the work seem to be balanced in presentation? | Yes |  |  |  |
| **Date** | Does the item have a clearly stated date related to content? No easily discernible date is a strong concern. | Yes |  |  |  |
|  | If no date is given, but can be closely ascertained, is there a valid reason for its absence? |  |  | N/A |  |
|  | Check the bibliography: have key contemporary material been included? |  |  | N/A |  |
| **Significance** | Is the item meaningful? (feasibility, utility and relevance) | Yes |  |  |  |
|  | Does it add context? | Yes |  |  |  |
|  | Does it enrich or add something unique to the research? | Yes |  |  |  |
|  | Does it strengthen or refute a current position? | Yes |  |  |  |
|  | Would the research area be lesser without it? | Yes |  |  |  |
|  | Is it integral, representative, typical? | Yes |  |  |  |
|  | Does it have impact? (in the sense of influencing the work or behavior of others) | Yes |  |  |  |
